# Psychometric Properties of the Questionnaire of Psychosocial Factors in University Environments

**DOI:** 10.3390/bs16060891

**Published:** 2026-06-01

**Authors:** Irene Margarita Espinosa Parra, Rodrigo Vargas Salomón, Edtna Elvira Jáuregui Ulloa, Elba Díaz Toro, Antonio Kobayashi Gutiérrez

**Affiliations:** 1 Department of Health Psychology, University Center for Health Sciences, University of Guadalajara, Guadalajara 44340, Mexico; irene.parra@uan.edu.mx; 2 Academic Program of Psychology, Autonomous University of Nayarit, Tepic 63000, Mexico; 3 Department of Health Sciences, University Center of Los Altos (CUAltos), University of Guadalajara, Tepatitlán de Morelos 47620, Mexico; 4 Department of Human Movement Sciences, University Center for Health Sciences, University of Guadalajara, Guadalajara 44340, Mexico; edtna.jauregui@academicos.udg.mx; 5 Comprehensive Cancer Center Hospital, School of Dental Medicine, University of Puerto Rico, San Juan 365067, Puerto Rico; elba.diaz@upr.edu; 6 Department of Neuroscience, Western Biomedical Research Center, IMSS, Guadalajara 44340, Mexico; drkoba@hotmail.com

**Keywords:** psychosocial factors, protective factors, university students, questionnaire development, reliability, scale validation, factor analysis

## Abstract

Psychosocial factors are associated with the well-being of university students, influencing their academic demands, degree of autonomy and control, and perceived support within their learning environment. Based on the demand–control–support model, the Questionnaire of Psychosocial Factors in University Environments (CFPAU, for its acronym in Spanish) was developed. This newly created instrument was designed to assess risk and protective factors in university students. This study included a total sample of 1221 Mexican students from two public universities in Mexico. The samples were randomly divided into two equivalent groups. Exploratory factor analysis (EFA) was performed on the first group (n_1_ = 611), and confirmatory factor analysis (CFA) was performed on the second group (n_2_ = 610) via the diagonally weighted least squares (DWLS) method. The final structure consisted of 5 global dimensions and 13 specific subscales, including psychological demands, active study and professional development opportunities, institutional quality and social relationships, recognition and career certainty, and school–life conflict. The CFA results showed adequate fit across the five dimensional models (CFI range = 0.921–1.00; TLI range = 0.910–1.00; RMSEA range = 0.000–0.065; SRMR range = 0.003–0.072). Factorial invariance by sex showed stability in the configural, metric, and scalar models, and subscale reliability was adequate (α and ω = 0.71–0.90). Furthermore, convergent and divergent validity were verified through correlations in the expected direction via the WHO-5 and DASS-21. These findings support the structural validity and internal consistency of the CFPAU, confirming its usefulness in identifying psychosocial risk and protective factors in university students.

## 1. Introduction

The university stage involves multiple changes, new academic contexts, the establishment of new social relationships and, in some cases, independence from family. This transition from high school to university requires students to develop greater levels of self-sufficiency, which leads to changes in their lifestyle (Chacón Cuberos et al., 2017; Pierella, 2019; Duche Pérez et al., 2020; Salazar, 2024). Given its close relationship with psychosocial factors, adaptation to this academic dynamic can directly impact a student’s overall well-being.

Psychosocial factors are closely related to academic stress, which is defined as the emotional, cognitive, and behavioral response to the demands and pressures arising from the educational process (Escobar Zurita et al., 2018). This stress is mediated by the interaction between individual phenomena (emotions, coping skills, and perceptions of stress) and contextual phenomena (academic demands and interpersonal relationships), which influence students’ educational trajectories and well-being.

Several studies have documented the psychosocial risks associated with university life, such as academic work overload, uncertainty about the professional future, imbalance between academic demands and personal life, economic insecurity, and a lack of support networks (Carranza-Esteban et al., 2017; Tafani et al., 2013; Estrada-Araoz et al., 2024). These situations can contribute to the development of symptoms such as depression, anxiety, or academic fatigue, understood as emotional exhaustion resulting from the demands of the university environment (Zhang et al., 2025).

Recent studies have consistently documented the multidimensional nature of psychosocial factors in university environments. Academic workload, assessment pressure, and difficulty in reconciling academic and personal life have been identified as primary stressors affecting students’ physical and psychological well-being (Quezada Díaz et al., 2024; Martínez-Torteya et al., 2023). Furthermore, institutional factors such as sense of belonging, perceived social support, and role clarity have been recognized as key protective resources that buffer the impact of academic demands on mental health (Curelaru & Curelaru, 2024; Palmieri et al., 2023). These findings underscore the need for comprehensive assessment instruments that are capable of simultaneously capturing both risk and protective psychosocial factors in university populations, particularly in Latin American contexts where validated tools remain scarce.

Previous international and national studies have suggested that the university stage represents a period characterized by exposure to multiple academic and psychosocial stressors that may impact students’ mental health. Research has particularly highlighted anxiety, depression, and suicidal behavior as some of the most serious problems reported among university populations (Jones & Essounga, 2024; López-Ramírez et al., 2024; Eisenberg et al., 2023; Liu et al., 2019).

In contrast, protective factors such as social support, a sense of belonging, institutional recognition, and opportunities for personal and professional development can act as buffers, reducing the impact of risks and promoting adaptation to the university environment (Jenson & Fraser, 2021; Aduviri, 2025).

The demand–control–social support conceptual model, proposed by Karasek (1979) and Johnson and Hall (1988), allows for an understanding of the interactions amongst demands, resources, and support. According to this model, prolonged exposure to high academic demands with little control over one’s own learning process and insufficient social support increases the risk of psychological distress, whereas having these resources in a balanced way promotes well-being.

The demand–control–social support theoretical framework has been widely validated using existing instruments such as the COPSOQ (Copenhagen Psychosocial Questionnaire) and the SUSESO/ISTAS21 (Social Security Superintendency (SUSESO [Superintendencia de Seguridad Social] in Spanish), in collaboration with the Spanish Trade Union Institute for Work, Environment, and Health (ISTAS [Instituto Sindical de Trabajo, Ambiente y Salud] in Spanish)), which were designed primarily for work and organizational settings. While adaptable, these tools fail to capture the unique psychosocial demands of the university context, such as pressure for academic excellence, career uncertainty, and school–life conflict (interference between academic demands and personal/family life). Therefore, a measurement instrument that, based on this theoretical model, integrates the specific characteristics of the student experience is needed, thus providing a more precise tool for identifying risks and resources within this population.

From this perspective, the Questionnaire of Psychosocial Factors in University Environments (CFPAU), specifically designed for the Mexican academic context, was developed. The instrument integrates risk and protective factors and is structured in five dimensions—(1) psychological demands, (2) active work and possibilities for professional development, (3) institutional quality and social relationships, (4) recognition and certainty in the career, and (5) school–life conflict—maintaining coherence with the model and reflecting its fundamental components: academic demands, control over the formative process, and social support perceived by students (Karasek, 1979; Johnson & Hall, 1988).

Therefore, the CFPAU enables differential identification of the quantitative, cognitive, sensory, and emotional psychological demands that students face, as well as the potential incompatibility of these demands with other needs in their personal or professional lives. It also assesses perceived autonomy, opportunities for development, the quality of institutional relationships, and the level of recognition received from the university.

From a psychosocial perspective, this model allows us to understand how subjectivities are constructed within the social context and how they influence interpersonal relationships and academic trajectories (Bonvillani, 2023). As Estrada-Araoz et al. (2024) noted, psychosocial factors are key to adequate academic development, as they directly impact students’ psychological well-being.

In this sense, González-Peiteado et al. (2017) indicated that one way to evaluate quality in higher education institutions is through their students who, as beneficiaries of academic training, reflect their satisfaction through motivation, commitment, and sense of belonging.

Therefore, the aim of this study was to develop and evaluate the psychometric properties of the CFPAU, a novel instrument designed specifically for university students that integrates risk and protective factors. The following hypotheses are proposed: (1) the CFPAU presents a multidimensional structure consistent with the theoretical framework; (2) it exhibits adequate model-fit indices in the confirmatory factor analysis (CFA) (CFI > 0.90; RMSEA < 0.08); and (3) it demonstrates satisfactory levels of internal consistency (α and ω > 0.70).

## 2. Methods and Materials

### 2.1. Participants

A convenience sampling method was used to recruit undergraduate students from two higher education institutions in Mexico: the University Center of Los Altos of the University of Guadalajara (CUALTOS) and the Autonomous University of Nayarit (UAN). The sample consisted of 1221 university students, of whom 352 (28.8%) were from CUALTOS and 869 (71.2%) were from UAN. A total of 1243 questionnaires were administered, 22 of which were eliminated due to a lack of consent or inconsistent responses, leaving 1221 valid instruments for analysis. The larger proportion of participants from UAN is explained by its institutional size, which is approximately three times larger than that of CUALTOS. Students of both sexes from diverse academic programs and semesters were included, ensuring sample heterogeneity and institutional representativeness. Representativeness was achieved across more than 90% of the educational programs at both institutions and across most academic semesters.

Convenience sampling was selected, as it is a widely accepted approach in the initial development and psychometric validation of measurement instruments, enabling the efficient recruitment of large and heterogeneous samples necessary for exploratory and confirmatory factor analyses (Clark & Watson, 1995, 2019; Tabachnick & Fidell, 2013; Nunnally & Bernstein, 1994; Schreiber, 2021). This method has been consistently employed in analogous scale development studies within university populations.

Sociodemographic data. For this study, an ad hoc survey was designed to obtain relevant information on sex, age, university, major, semester, and type of study, selected for their relevance as intervening variables.

Questionnaire of Psychosocial Factors in University Environments (CFPAU). The CFPAU was explicitly designed to assess psychosocial risk and protective factors in university students on the basis of the demand–control–social support model (Karasek, 1979; Johnson & Hall, 1988). Its design referenced the conceptual and methodological structure of the Copenhagen Psychosocial Questionnaire (COPSOQ), which was developed in 2000 by Kristensen and Borg and subsequently distributed by the COPSOQ International Network (Red Internacional COPSOQ, 2020). It also referenced the Spanish-language versions, CoPsoQ-ISTAS21 (S. Moncada et al., 2004, 2005, 2014; L. S. Moncada et al., 2021), and the Chilean version SUSESO/ISTAS21 (Superintendencia de Seguridad Social del Gobierno de Chile, 2020), a set of instruments that assess psychosocial risk in the workplace. The original CFPAU items were developed through a theoretically guided adaptation of COPSOQ domains to university-specific psychosocial experiences, including academic demands, perceived control, institutional support, recognition, career certainty, and school–life conflict.

The CFPAU instrument consists of Likert-type items with five response options, reflecting the degree of presence of each psychosocial factor, both risk and protective, in the context of university life. The CFPAU considers five theoretical dimensions:

1. Psychological Demands;
2. Active Study and Opportunities for Professional Development;
3. Institutional Quality and Social Relationships;
4. Recognition and Certainty in the Career;
5. School–Life Conflict.

For more information on these dimensions, please consult the Supplementary Materials. The CFPAU was originally developed and administered in Spanish. The English version included in the Supplementary Materials corresponds to a translation prepared for publication purposes.

### 2.2. Procedure

Data were collected at two public universities in Mexico, the Autonomous University of Nayarit (UAN) and the University Center of Los Altos (CUALTOS) of the University of Guadalajara, from September–December 2024.

All the participants were volunteers and received no compensation. The instruments were administered electronically via a Google Form in accordance with the CHERRIES checklist, which included an informed consent request clarifying the voluntary nature of participation, the objectives and purpose of the research, the protection of anonymity, the right to stop answering the questionnaire if they wished, and the explicit clarification that the data collected would be used exclusively for this research.

SPSS v.23.0 and the R statistical environment v.4.4.1 (R Core Team, 2024) with the RStudio software (RStudio Team, 2026.01.0+392)—specifically the psych package v 2.5.6 (Revelle, 2023) and laavan v 0.6-21—were used for exploratory factor analysis (EFA) and confirmatory factor analysis (CFA).

### 2.3. External Instruments

WHO-5 Well-Being Index

The World Health Organization Well-Being Index (WHO-5) is a brief self-report instrument designed to assess subjective psychological well-being. It consists of five items that evaluate positive emotional states experienced during the previous two weeks. Responses are rated on a 6-point Likert scale ranging from 0 (“at no time”) to 5 (“all of the time”), with higher scores indicating greater well-being. The WHO-5 has shown adequate reliability and validity in diverse populations, including university students (Topp et al., 2015; World Health Organization, 1998).

Depression, Anxiety, and Stress Scale (DASS-21)

The Depression, Anxiety, and Stress Scale (DASS-21) is a self-report instrument designed to assess negative emotional states. It consists of 21 items distributed across 3 subscales—depression, anxiety, and stress—with 7 items per dimension. The depression subscale evaluates symptoms such as dysphoria, hopelessness, and lack of interest; the anxiety subscale assesses physiological arousal and situational anxiety; and the stress subscale measures tension and irritability. Items are rated on a 4-point Likert scale ranging from 0 (“did not apply to me at all”) to 3 (“applied to me most of the time”) (Lovibond & Lovibond, 1995). The DASS-21 has demonstrated adequate psychometric properties, including strong internal consistency and construct validity, across diverse populations including university students (Henry & Crawford, 2005; Antony et al., 1998; Román et al., 2016).

### 2.4. Statistical Analysis

To explore the factor structure of the CFPAU, the total sample was randomly divided into two equivalent groups (n_1_ = 611; n_2_ = 610). Given the multidimensional and theoretically grounded structure of the CFPAU, EFAs were conducted within each of the five a priori dimensions rather than on the full item pool. This decision was based on three considerations: (1) the five dimensions of the CFPAU are theoretically distinct and derived from well-differentiated components of the demand–control–social support model (Karasek, 1979; Johnson & Hall, 1988); (2) conducting a single EFA on all 74 items would produce an overly complex factor solution that could obscure theoretically meaningful within-dimension structure (Matsunaga, 2010); and (3) domain-specific EFAs are consistent with the validation approach used in the reference instruments on which the CFPAU is based, such as the COPSOQ (Kristensen et al., 2005). A full-scale EFA was considered but deemed inappropriate, given the theoretical distinctiveness of the dimensions and the risk of factorial indeterminacy with a large item pool.

A parallel analysis based on 1000 randomly generated correlation matrices and an exploratory factor analysis (EFA) were performed with the first group (n_1_ = 611). Given the ordinal nature of the Likert-type response scale, polychoric correlations were used as the input matrix, and the weighted least squares (WLS) extraction method with oblimin rotation was applied, which is suitable for correlated factor structures in ordinal data (Fabrigar et al., 1999; Schreiber, 2021). Factor loadings ≥ 0.40 were used as the criterion for item retention, and items that cross-loaded on multiple factors or failed to meet this threshold were eliminated. The internal consistency of each factor was estimated using Cronbach’s α coefficient and McDonald’s ω coefficient, calculated with the psych package in R (Revelle, 2023).

Subsequently, confirmatory factor analyses (CFAs) were performed with the second group (n_2_ = 610) via the diagonally weighted least squares (DWLS) method. The five-dimensional, 13-factor structure from the exploratory factor analysis (EFA) was used, and the factor with the highest conceptual coherence and best fit indices was selected. The χ^2^/df, RMSEA, SRMR, CFI, and TLI indicators were evaluated, using reference values of CFI and TLI ≥ 0.90, RMSEA ≤ 0.08, and SRMR ≤ 0.08 (Hu & Bentler, 1999). Modifications were applied only when there was theoretical support and statistical evidence, as indicated by the modification indices.

Convergent and divergent validity were analyzed via Spearman correlations between the dimensions of the CFPAU and two previously validated instruments (WHO-5: α = 0.904, ω = 0.904; DASS-21: α = 0.956, ω = 0.956): the Depression, Anxiety and Stress Scale (DASS-21; Lovibond & Lovibond, 1995), which measures negative emotional states and was used as evidence of convergent validity; and the WHO Well-Being Index (WHO-5; Cornelio & Contreras, 2020), which assesses subjective well-being and was used as evidence of divergent validity, given that it represents a conceptually distinct but theoretically related construct.

Finally, measurement invariance by sex was evaluated using the DWLS estimation method within the lavaan package in R (Rosseel, 2012), applied to the confirmatory sample (n_2_ = 610; men = 249, 40.8%; women = 355, 58.2%; unspecified = 6, 1.0%). Three hierarchical levels of invariance were tested sequentially: configural invariance (same factor structure across groups), metric invariance (equal factor loadings), and scalar invariance (equal item intercepts). Model comparisons were based on changes in fit indices: ΔCFI < 0.010, ΔTLI < 0.010, and ΔRMSEA < 0.015, following the criteria of Cheung and Rensvold (2002). The Δχ^2^ test was also reported, although given its sensitivity to sample size, decisions were primarily based on changes in CFI, TLI, and RMSEA.

## 3. Results

The participants’ ages ranged from 17 to 72 years, with a mean of 20.61 years (SD = 3.53), a median of 20, and a mode of 20, indicating a wide range with few outliers. With respect to gender, 58.6% were female, 40.5% were male, and 1.3% preferred not to specify. Representativeness was achieved in more than 90% of the educational programs at both institutions and across most academic semesters, a relevant aspect since some items on the CFPAU (Center for Academic Performance Assessment) may vary depending on academic progress. Furthermore, the vast majority of the students (98.6%) were enrolled in on-campus programs, whereas 1.39% were enrolled in blended learning programs.

All participants were enrolled in undergraduate (bachelor’s degree) programs. The wide age range observed (17–72 years) is explained by the presence of a small number of non-traditional students who pursue higher education at a later stage in life, a phenomenon documented in Mexican public universities (mean age = 20.61, SD = 3.53, median = 20), where the distribution confirms that the vast majority of participants were of traditional university age.

Descriptive statistics were computed for all items, and distributional properties were evaluated using skewness and kurtosis indices based on thresholds proposed by Hair et al. (2010). Although Likert-type responses are ordinal, this assessment supported the subsequent modeling approaches. Item-level frequency distributions and missing data percentages are reported in Supplementary Table S1, with no collapsed categories, as all original 5-point response options were retained.

### 3.1. Exploratory Factor Analysis

Exploratory factor analysis (EFA) was performed on the 74 original items of the Questionnaire of Psychosocial Factors in University Environments (CFPAU) in the first group of 611 participants (men = 241, 39.4%; women = 365, 59.7%; unspecified = 5, 0.8%), with a mean age of 20.56 ± 3.47 years (range 17–69). The majority of students belonged to the Autonomous University of Nayarit (72.3%), while the remaining belonged to the University Center of Los Altos (27.7%).

Data showed adequate overall internal consistency (α = 0.883; ω = 0.900); the percentage of respondents with the minimum score was 0.3%. Factor extraction was performed via the weighted least squares (WLS) method for each dimension (Schreiber, 2021). Sample adequacy was satisfactory (KMO = 0.900; Bartlett, *p* < 0.001). The number of factors to retain was determined through parallel analysis based on 1000 random correlation matrices. Factor retention was decided by comparing the observed eigenvalues with those obtained from the simulated data, retaining only those factors whose observed eigenvalues exceeded the corresponding random eigenvalues.

In Dimension 1, parallel analysis suggested four factors. Factor one explains 19% of the variance and corresponds to emotional labor (items 10, 11, 12, 13, and 14). Factor two explains 18% of the variance and arises from the fusion of quantitative and cognitive psychological demands (items 1, 2, 3, 4, 6, and 7). Factor three explains 15% of the variance and corresponds to sensory demands (items 16, 17, 18). Factor four included only one item and was therefore eliminated. Items 5, 8, 9, 15, and 19 were also discarded because they did not belong to the corresponding factor.

In Dimension 2, the parallel analysis suggested five factors, whereas the original instrument had three factors. Two exploratory factor analyses (EFAs) were performed. The five-factor model (RMSEA = 0.081; RMSR = 0.04) and the three-factor model (RMSEA = 0.085; RMSR = 0.06) yielded similar indices; the three-factor model was retained due to its greater theoretical coherence. The results revealed factor 1 (15% of the variance) as professional sense and commitment (items 30, 31, 32, 33, 34, 35, 36); factor 2 (11%) as control over work time at the university (items 20, 21, 22, 23, 24, 25, 26, 27, 28, 29); and factor 3 (9%) as integration into the university (items 37, 38, 39, 40).

In Dimension 3, parallel analysis suggested four factors compared with the original five factors. Both models showed similar fits (four factors: RMSEA = 0.066; RMSR = 0.03; 5 factors: RMSEA = 0.065; RMSR = 0.03), so the structure closest to the original was retained. Factor 1 (19%) corresponded to institutional quality (items 50, 52, 58–62); factor 2 (12%) corresponded to a sense of belonging (items 51, 54–57); factor 3 (12%) corresponded to predictability and role clarity (items 41–45); and factor 4 (8%) corresponded to role conflict (items 46–49). The fifth factor explained only 3% of the variance and contained a single item (item 53), so it was eliminated. The reinforcement factor and social support at the university were represented within institutional quality and sense of belonging.

In Dimension 4, parallel analysis suggested three factors, whereas the original instrument proposed two factors. The three-factor model (RMSEA = 0.061; RMSR = 0.02) provided a better fit than the two-factor model (RMSEA = 0.121; RMSR = 0.05); however, given its theoretical consistency, the two-factor structure was retained. Factor 1 (29%) corresponded to insecurity in university studies (items 66–70), and factor 2 (22%) corresponded to recognition (items 63–65).

In Dimension 5, parallel analysis indicated a one-factor solution. EFA showed an RMSR = 0.02 and an RMSEA = 0.123; the single factor explained 6.5% of the variance and was identified as school–life conflict (items 71–74). Although the RMSEA was higher than recommended, the factor was retained because of its theoretical relevance and adequate internal consistency (α = 0.861; ω = 0.869).

Based on the EFA, the final dimensional structure of the CFPAU was defined (Table 1), consisting of 5 global dimensions and 13 specific subscales. Internal consistency was estimated separately for each subscale using Cronbach’s alpha and McDonald’s omega:

1. Psychological demands: (a) quantitative and cognitive demands (6 items), (b) emotional labor (5 items), and (c) sensory demands (3 items).
2. Active work and professional development opportunities: (a) control over work time at the university (10 items), (b) professional sense and commitment (7 items) and (c) integration into the university (4 items).
3. Institutional quality and social relations: (a) predictability and clarity of role (5 items), (b) role conflict (4 items), (c) sense of belonging (5 items) and (d) institutional quality (7 items).
4. Recognition and certainty in one’s career: (a) recognition (3 items) and (b) insecurity in university studies (5 items).
5. School–life conflict: a single factor of 4 items.

**Table 1:** Dimensional structure of the CFPAU structure proposed by the EFA.

Dimensions and Factors of the CFPAU	No. Items	α	ω
Dimension 1: Psychological demands			
1.1 Quantitative and cognitive psychological demands *	6	0.848	0.844
1.2 Emotional work *	5	0.872	0.870
1.3 Sensory psychological demands *	3	0.827	0.828
Dimension 2: Active work and opportunities for professional development			
2.4 Control over working hours at the university	10	0.747	0.727
2.5 Professional purpose and commitment	7	0.808	0.810
2.6 Integration into the university	4	0.709	0.698
Dimension 3: Institutional quality and social relations			
3.7 Predictability and clarity of roles	5	0.815	0.811
3.8 Role conflict *	4	0.756	0.751
3.9 Sense of belonging	5	0.841	0.845
3.10 Institutional quality	7	0.885	0.886
Dimension 4: Recognition and certainty in the career			
4.11 Recognition	3	0.798	0.800
4.12 Insecurity in university studies *	5	0.810	0.804
Dimension 5: School–life conflict			
5.13 Conflict between school and life *	4	0.861	0.869

Note: The 6 factors marked with an asterisk (*) are theoretically considered risk factors, while the remaining 7 factors are considered protective factors. This clarification is important for interpreting the instrument and was taken into account in the internal consistency calculations.

### 3.2. Confirmatory Factor Analysis

A confirmatory factor analysis (CFA) was performed with the second sample group (n_2_ = 610) to evaluate the adequacy of the 5-dimensional, 13-factor structure identified in the EFA. Since the items were measured on an ordinal Likert scale, the diagonally weighted least squares (DWLS) estimation method, which is considered appropriate for this type of data (Mindrila, 2010), was used. The analyses were performed in the R environment (R Core Team, 2024) via the RStudio software (RStudio Team, 2024) and the lavaan statistical package (Rosseel, 2012). Error covariances were added only when modification indices exceeded 10 and when items showed semantic similarity, in order to improve model fit while avoiding overfitting. These modifications were minimal and theoretically justified.

**Figure 1:**
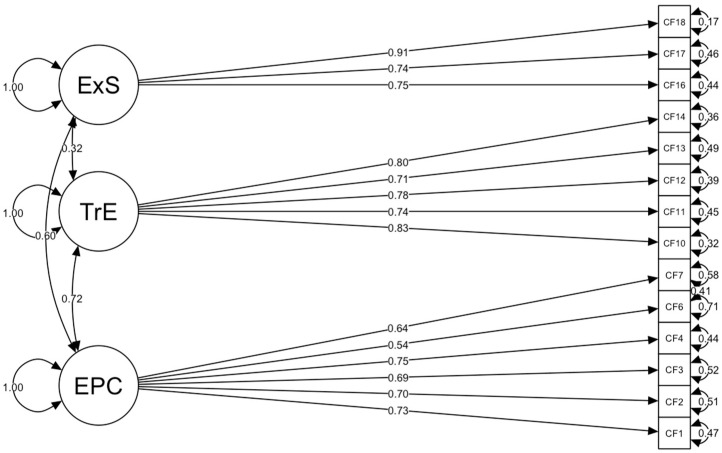
CFA of Dimension 1: psychological demands.

**Figure 2:**
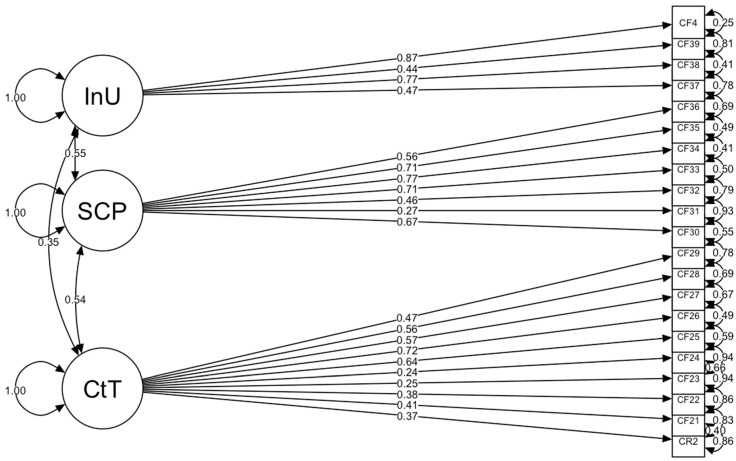
Modified model of active study and opportunities for professional development.

**Figure 3:**
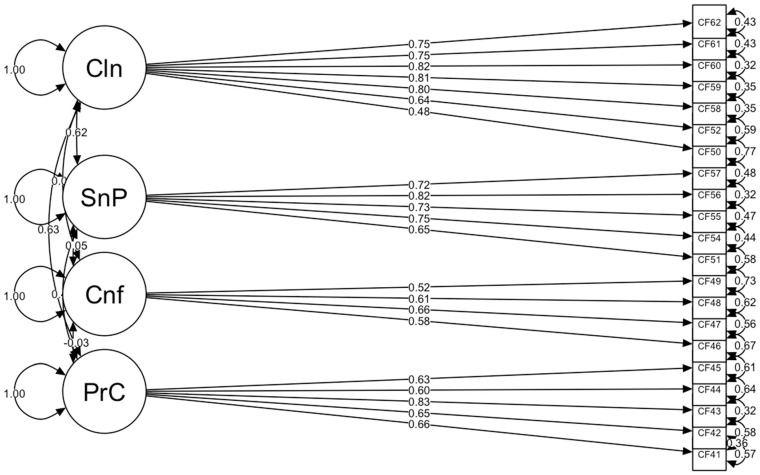
Modified model of institutional quality and social relationships.

**Figure 4:**
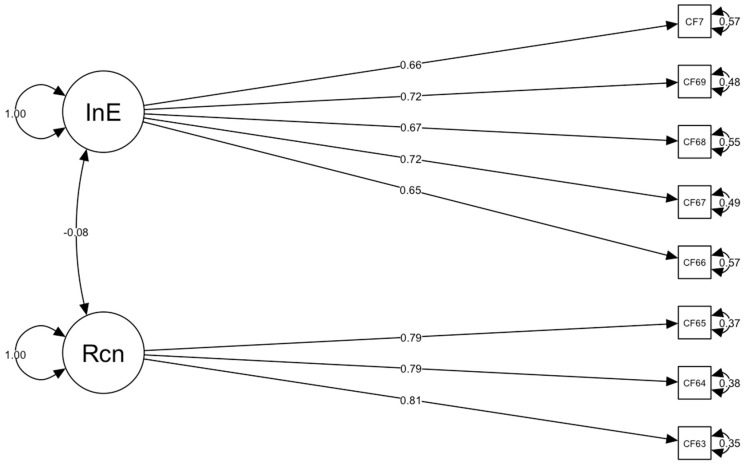
Recognition and career certainty model.

**Figure 5:**
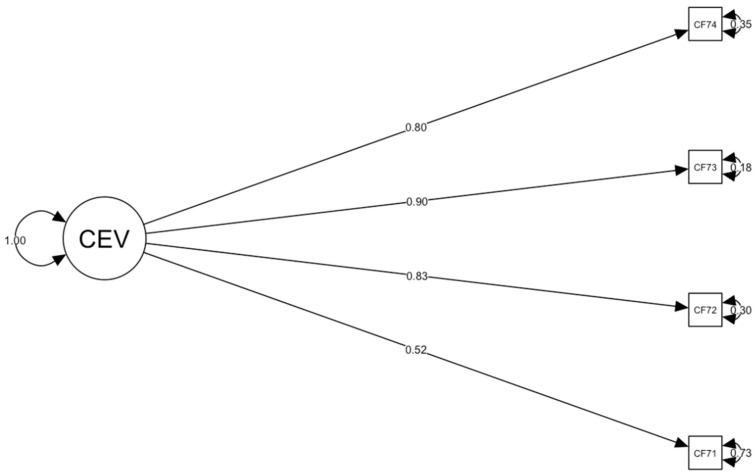
School–life conflict model.

Table 2 presents the fit indices obtained for the five confirmatory models of the CFPAU. Given the domain-specific validation approach, fit indices are reported separately for each dimensional model. A global CFA with all 68 items and 13 factors simultaneously was considered but deemed inappropriate given the large number of parameters relative to the sample size (n_2_ = 610) and the theoretical rationale for conducting analyses within each dimension. In general, the CFI and TLI values were above 0.90, and the RMSEA and SRMR error indices were below 0.08, indicating an adequate overall fit of the model. The modifications made were minimal and theoretically justified, confirming the structural validity of the instrument. Item content is provided in Supplementary Materials, whereas Figure 1, Figure 2, Figure 3, Figure 4 and Figure 5 visually represent the corresponding CFA models and the relationships between latent factors and their indicators.

For Dimension 1, the initial model showed an acceptable fit (χ^2^/df = 213.66/74, RMSEA = 0.056, SRMR = 0.063, CFI = 0.983, TLI = 0.979), and the covariance between items CF6 and CF7 slightly improved the fit (χ^2^/df = 182.07/73, RMSEA = 0.050, SRMR = 0.058, CFI = 0.987, TLI = 0.984), confirming its correspondence with the demand component of the demand–control–social support model.

This dimension comprises three factors—(1) sensory demands (ExS); (2) emotional labor (TrE); and (3) quantitative–cognitive psychological demands (EPC)—which showed positive and significant covariances, demonstrating the expected interrelation between the components of the construct (Figure 1).

In Dimension 2, representing active work and professional development opportunities, a reasonable fit was observed (χ^2^/df = 903.91/186, RMSEA = 0.080, SRMR = 0.085, CFI = 0.881, TLI = 0.866). Covariances were allowed between items with high semantic interdependence (CF20–CF21, CF23–CF24). Moderate to high factor loadings support the theoretical coherence with the control component, representing students’ perceptions of autonomy and control over their training process and academic development opportunities, which is consistent with the model.

This dimension is composed of three factors: (1) control over working time at the university (CtT); (2) professional sense and commitment (SCP); (3) integration into the university (InU) (Figure 2).

For Dimension 3, representing institutional quality and social relationships, the results showed a good fit and consistent internal relationships (χ^2^/df = 415.68/182, RMSEA = 0.046, SRMR = 0.061, CFI = 0.975, TLI = 0.971). This dimension is composed of four factors: role predictability and clarity (PrC), role conflict (Cnf), sense of belonging (SnP), and institutional quality (Cln). A negative covariance was observed between PrC and Cnf, which is theoretically consistent with the idea that greater role clarity is associated with a lower perception of conflict. A relationship was also found between a sense of belonging, institutional support, and role predictability and clarity, which constitutes a protective psychosocial resource that facilitates adaptation to the university environment, reflecting the social support component of the DCS (Figure 3).

Dimension 4, representing recognition and career certainty, had excellent fit indices without requiring modifications (χ^2^/df = 34.76/19, RMSEA = 0.037, SRMR = 0.046, CFI = 0.989, TLI = 0.984). This dimension is composed of two factors—(1) recognition (Rcn) and (2) insecurity in university studies (InE)—both with satisfactory factor loadings.

A negative covariance was observed between the factors, which confirms their theoretical independence, recognition as a protective factor linked to institutional support, and insecurity as a risk factor linked to uncertainty regarding the university trajectory (Figure 4).

Finally, Dimension 5, representing school–life conflict, showed a perfect fit and a stable unidimensional model (χ^2^/df = 0.043/2, RMSEA = 0.000, SRMR = 0.003, CFI = 1.00, TLI = 1.00), which supports the theoretical and empirical consistency, robustness and unidimensionality of this construct (Figure 5). This finding demonstrates that academic demands significantly interfere with personal and social life, which is consistent with the demand component.

To further examine the robustness of the model, a sensitivity analysis was conducted by re-estimating the CFA for Dimension 2 after removing items with standardized factor loadings below 0.40. The reduced model showed a modest improvement in fit indices (CFI = 0.949, TLI = 0.939, RMSEA = 0.062, SRMR = 0.068) compared to the original model (CFI = 0.921, TLI = 0.910, RMSEA = 0.065, SRMR = 0.072). However, this improvement did not substantially alter the overall interpretation of the construct. Given the theoretical relevance of the retained items and their contribution to content validity, the original model specification was maintained.

Overall, the CFA results confirm that the CFPAU has a stable multidimensional structure; all five dimensions achieved fit indices within acceptable ranges after minimal and theoretically justified modifications. This structure confirms the instrument’s coherence with the demand–control–social support model, reflecting a balance between risk and protective factors in the university setting.

### 3.3. Convergent and Divergent Validity

According to the demand–control–social support model, psychosocial environments characterized by high demands and limited control or social support are associated with greater psychological distress and lower levels of subjective well-being. Therefore, correlations between the CFPAU dimensions and measures of psychological distress (DASS-21) and subjective well-being (WHO-5) were expected in theoretically consistent directions.

Based on this theoretical framework, once the factor structure and internal consistency of the CFPAU were confirmed, its convergent and divergent validity was evaluated through correlations with two external scales: the Subjective Well-Being Scale (WHO-5; Cornelio & Contreras, 2020) and the Depression, Anxiety, and Stress Scale (DASS-21; Lovibond & Lovibond, 1995).

Table 3 presents descriptive statistics and Pearson correlations among protective factors, risk factors, and the external validity measures (WHO-5 and DASS-21 subscales). Protective factors were operationalized as the mean score of the seven factors theoretically associated with resources and well-being, whereas risk factors were operationalized as the mean score of the six factors associated with demands and psychological distress, consistent with the demand–control–social support model (Karasek, 1979; Johnson & Hall, 1988).

Previous research has documented gender differences in the experience of academic stress and psychological distress among university students, with female students generally reporting higher levels of anxiety, stress, and lower levels of mental well-being compared with male students (Bayram & Bilgel, 2008; Zhu et al., 2025). Given these differences, it is important to evaluate measurement invariance across sex to ensure that the CFPAU assesses psychosocial factors equivalently in men and women.

### 3.4. Analysis of Invariance by Sex

A factorial invariance analysis by sex was performed on the adjusted 68-item CFPAU, which revealed that the model maintains structural stability at the configural, metric, and scalar levels across the 5 dimensions. The Δχ^2^ test was used, along with changes in the CFI (ΔCFI) with a critical significance level < 0.010 and a critical significance level of 0.010 for ΔTLI and 0.015 for ΔRMSEA (Cheung & Rensvold, 2002).

The factorial invariance analysis by sex for Dimension 1 showed significant differences between the configural and metric models (Δχ^2^ = 81.48, *p* < 0.001), as well as between the metric and scalar models (Δχ^2^ = 29.91, *p* = 0.0016). However, the changes observed in the fit indices (ΔCFI = 0.0043; ΔTLI = 0.004; ΔRMSEA = 0.006 and 0.0001, respectively) remained within the acceptable thresholds proposed by Cheung and Rensvold (2002), which supports the metric and scalar invariance of the model for both groups.

For Dimension 2, the factorial invariance analysis by sex revealed significant differences between both the configural and metric models (Δχ^2^ = 47.12, *p* = 0.0002) and between the metric and scalar models (Δχ^2^ = 102.39, *p* < 0.001). However, the changes in the fit indices (ΔCFI = 0.0026; ΔTLI = −0.0004; ΔRMSEA = −0.0002 for the metric step, and ΔCFI = 0.0074; ΔTLI = 0.0047; ΔRMSEA = 0.0019 for the scalar step) remained within the recommended thresholds, thus supporting the metric and scalar invariance of the model between men and women.

For Dimension 3, the factorial invariance analysis by sex revealed significant differences between the configural and metric models (Δχ^2^ = 49.96, *p* < 0.001), as well as between the metric and scalar models (Δχ^2^ = 46.06, *p* < 0.001). However, the changes in the fit indices were minimal (ΔCFI = 0.0017 and 0.0015; ΔTLI = 0.0007 and 0.0004; ΔRMSEA = 0.0006 and 0.0003, respectively) and remained below the recommended thresholds. These results support the existence of metric and scalar invariance of the model between men and women.

For Dimension 4, the factorial invariance analysis by sex revealed no significant differences between the configural and metric models (Δχ^2^ = 3.13, *p* = 0.792), supporting metric invariance. Similarly, the comparison between the metric and scalar models also revealed no significant difference (Δχ^2^ = 11.78, *p* = 0.066), suggesting that scalar invariance is also present. Changes in the fit indices (ΔCFI = 0.0022; ΔTLI = 0.0010; ΔRMSEA = 0.0012) remained within the recommended limits, supporting the structural equivalence of the model between men and women.

For Dimension 5, the factorial invariance analysis by sex revealed no significant differences between the configural and metric models (Δχ^2^ = 0.34, *p* = 0.95), supporting metric invariance. However, the comparison between the metric and scalar models indicated a statistically significant difference (Δχ^2^ = 9.90, *p* = 0.010), which could call into question scalar invariance. Despite this, the changes in the fit indices were minimal (ΔCFI = 0.0008; ΔTLI = 0.0044), except for the RMSEA (ΔRMSEA = 0.0183), which slightly exceeded the suggested threshold (<0.015). These results suggest that scalar invariance could be considered partial, and caution is advised when comparing latent means between groups in this dimension.

Table 4 presents the sex-specific invariance data for each dimension. The configural and metric models showed no statistically significant differences in Δχ^2^ in most cases, supporting metric invariance. However, scalar models showed a significant Δχ^2^ and some changes in fit indices above the recommended thresholds, indicating partial scalar invariance. In summary, the 68-item CFPAU method shows metric invariance by sex.

## 4. Discussion

The results obtained confirm the hypotheses and support the robust psychometric soundness of the CFPAU. Altogether, the exploratory and confirmatory factor analyses verified a 5-dimensional, 13-factor structure with satisfactory fit indices, providing empirical support for the proposed theoretical model. The multidimensional structure fits coherently with the demand–control–social support theoretical model, showing adequate internal consistency across all dimensions. These findings confirm the relevance of the theoretical framework and the metric quality of the instrument, which represents an original contribution as the first questionnaire designed to assess psychosocial risk and protective factors in Mexican university students.

These findings align with those of previous studies that validated psychosocial assessment instruments in work and educational contexts (Kristensen et al., 2005; S. Moncada et al., 2014; González-Peiteado et al., 2017). The CFPAU broadens the perspective by simultaneously integrating risk factors (academic demands, job insecurity, school–life conflict) and protective factors (social support, sense of belonging, institutional recognition). This dual approach of protection and risk represents a significant theoretical advancement, as it allows for the identification of both conditions that generate academic risk and resources that promote psychological well-being and adaptation to the university environment. In addition, the near-zero correlation observed between protective and risk factors suggests that these dimensions operate as relatively independent constructs rather than opposite ends of a single continuum, indicating that both types of factors may coexist in the university context.

From a methodological standpoint, the use of confirmatory factor analysis (CFA) with diagonally weighted least squares (DWLS) estimation and the sex invariance test strengthen the evidence for the structural validity of the CFPAU. The fit indices were consistent with expected values for instruments of a complex and multidimensional nature (Morata-Ramírez et al., 2015). As Fabrigar et al. (1999) and Mavrou (2015) noted, the interpretation of fit indices should be based on both theoretical coherence and the parsimony of the model and not solely on rigid statistical criteria. In this sense, the adjustments applied were made in a theoretically sound manner, preserving the structure proposed by the demand–control–social support model.

Furthermore, the factorial invariance analyses by sex provide additional evidence of the stability of the CFPAU. The results show metric invariance across all five dimensions, indicating that the factors maintain the same structure and item relationships for men and women. Although the school–life conflict dimension showed partial scalar invariance, the changes in the fit indices were minimal, suggesting that the instrument is conceptually equivalent in both groups. Therefore, the CFPAU can be used to compare structural associations between men and women.

The results of convergent and divergent validity reinforce the external validity of the CFPAU, showing positive correlations with the WHO-5 and negative correlations with the DASS-21 in the expected direction. This suggests that the protective dimensions of the instrument (control, support, and recognition) are associated with greater well-being, whereas the risk dimensions (demands, insecurity, and school–life conflict) are linked to higher levels of psychological distress. These findings are consistent with the literature that highlights the importance of balancing demands and resources in preventing psychological distress (Jenson & Fraser, 2021; Estrada-Araoz et al., 2024).

In theoretical terms, the confirmed five-dimensional structure of the instrument reflects the components of the “demand–control–social support” model by Karasek (1979) and Johnson and Hall (1988). Dimensions 1 and 5 correspond to the demand component, Dimension 2 corresponds to control, and Dimensions 3 and 4 correspond to social support. This articulation demonstrates the interdependence between the conditions of the academic environment and the personal and institutional resources available to students. Furthermore, this instrument facilitates institutional evaluations of university well-being and the design of evidence-based psychosocial interventions.

These findings suggest that the demand–control–social support model, which has been historically applied in the workplace, is transferable to the university context, validating its usefulness for understanding the psychosocial processes that affect students. The empirical confirmation of this structure in university populations supports the relevance of applying this theoretical framework to educational settings, where academic demands, control over the learning process, and institutional support play similar roles to equivalent factors observed in workplace contexts.

Finally, the psychometric robustness and theoretical coherence of the CFPAU support its usefulness as a diagnostic and research tool in higher education contexts. Its application can contribute to identifying emerging risk factors, guiding preventive university mental health strategies focused on managing academic workload, strengthening a sense of belonging, promoting academic recognition, and fostering healthier academic environments.

### Strengths, Limitations, and Future Lines of Work

One of the main strengths of this study lies in the development of the Questionnaire of Psychosocial Factors in University Environments (CFPAU), an instrument that integrates psychosocial risk and protective factors in a balanced way among university students. This approach offers a more comprehensive view of the students’ psychosocial context, identifying both the elements that can generate vulnerability and those that promote psychological well-being and academic adjustment.

The factor analyses performed allowed the CFPAU to be reduced from 74 to 68 items while maintaining the 5 dimensions and adequate levels of internal consistency (α between 0.709 and 0.885; ω between 0.698 and 0.886) in all its factors. This process contributed to a better understanding of the theoretical relevance of the demand–control–support model (Karasek, 1979; Johnson & Hall, 1988) for the analysis of university environments.

From this perspective, the university is interpreted as an environment of dynamic and complex interactions in which academic demands, perceived control, and real or perceived social support jointly influence students’ well-being and mental health. Thus, the CFPAU offers a tool to understand these interactions and guide preventive interventions and the promotion of psychosocial health.

Among the limitations of this study is the use of self-report measures, which can introduce biases stemming from social desirability or the subjective interpretation of the items. Likewise, the cross-sectional design and the use of nonprobability convenience sampling limit the possibility of establishing causal relationships and generalizing the results. In addition, the sample was drawn from only two public universities and included a greater proportion of students from the Autonomous University of Nayarit, which may restrict the representativeness of the findings. Although convenience sampling limits the generalizability of findings to broader university populations, this approach is consistent with standard practice in first-stage psychometric validation studies (Clark & Watson, 1995; Nunnally & Bernstein, 1994).

Another limitation concerns the length of the instrument. Although the refinement process reduced the CFPAU from 74 to 68 items, its administration may represent a response burden for participants, potentially affecting data quality through fatigue effects. Future studies should examine the feasibility of developing a short form of the CFPAU that preserves its multidimensional structure while reducing administration time, as has been achieved with similar instruments such as the COPSOQ short version (Kristensen et al., 2005).

Future research should replicate and expand the validation of the CFPAU in public and private higher education institutions, as well as in different cultural contexts in Latin America, using probabilistic sampling strategies, with the aim of strengthening its cross-cultural validity. Longitudinal and predictive studies are also suggested to examine the relationship of the CFPAU with indicators of subjective well-being, mental health, and academic performance, consolidating its contribution to health psychology.

## 5. Conclusions

The Questionnaire of Psychosocial Factors in University Environments (CFPAU) is a psychometrically sound tool for the integrated assessment of psychosocial risk and protective factors in university students, which is consistent with the demand–control–support model. Its multidimensional structure and internal consistency confirm its validity as a scientific instrument and its relevance as an applied tool.

The application of the CFPAU is fundamental for the design of institutional policies and preventive strategies in university mental health, as it allows for the identification of vulnerabilities, such as academic overload or career uncertainty, while simultaneously recognizing protective factors such as social support, a sense of belonging, and institutional recognition. From this perspective, the instrument contributes to both academic research and institutional practice aimed at promoting holistic well-being in higher education.

This study’s findings reinforce the idea that the university stage represents a critical period in youth development characterized by academic, social, and personal demands that directly impact psychological well-being. In this context, universities are responsible for implementing policies and programs that promote equity, sustainability, and the protection of students’ psychosocial health.

Beyond its diagnostic application, the CFPAU offers strategic indicators for designing interventions that promote socioemotional skills, healthy lifestyles, and institutional support networks. Its validation provides a solid foundation for advancing research and institutional practice in university mental health. Together, these elements position the CFPAU as an innovative tool for understanding and promoting well-being in university settings.

Furthermore, for the first time, the CFPAU enables a comprehensive, differentiated, and contextualized evaluation of university psychosocial factors in Mexico, reinforcing its potential impact on both research and the design of institutional policies.

## Figures and Tables

**Table 2:** Values obtained through CFA by dimension.

Dimensions of the CFPAU	Model	χ^2^/gL	RMSEA	SRMR	CFI	TLI
1. Psychological demands	**Initial model**	213.66/74	0.056	0.063	0.983	0.979
	**Modified model** (Covariance CF6 with CF7)	182.07/73	0.050	0.058	0.987	0.984
2. Active work and opportunities for professional development	**Initial model**	903.91/186	0.080	0.085	0.881	0.866
	**Modified model** (Covariance CF20–CF21 and CF23–CF24)	660.81/184	0.065	0.072	0.921	0.910
3. Institutional quality and social relations	**Initial model**	430.29/183	0.047	0.062	0.973	0.969
	**Modified model** (Covariance CF41–CF42)	415.68/182	0.046	0.061	0.975	0.971
4. Recognition and certainty in the career	**Initial model**	34.76/19	0.037	0.046	0.989	0.984
5. School–life conflict	**Initial model**	0.043/2	0.000	0.003	1.00	1.00

Note. The modifications indicated in the models refer to the changes made to the 74-item instrument used for the AFC.

**Table 3:** Spearman Correlations with the Subjective Well-Being Scale and Depression, Anxiety, and Stress Scale.

	M + SD	1	2	3	4	5
1. Protective factors	65.82 ± 13.04	--				
2. Risk factors	54.12 ± 9.81	0.005	--			
3. WHO-5 (Well-Being)	58.53 ± 21.73	0.329 **	−0.230 **	--		
4. Stress	17.98 ± 11.102	−0.187 **	0.287 **	−0.578 **	--	
5. Anxiety	13.35 ± 10.7	−0.144 **	0.254 **	−0.414 **	0.800 **	--
6. Depression	14.16 ± 10.9	−0.188 **	0.262 **	−0.528 **	0.811 **	0.795 **

Note. M = mean; SD = standard deviation. Correlations are Spearman. Protective factor score was computed as the mean of the following seven factors: control over working time at the university, professional sense and commitment, integration into the university, predictability and clarity of roles, sense of belonging, institutional quality, and recognition. Risk factor score was computed as the mean of the following six factors: quantitative and cognitive psychological demands, emotional labor, sensory psychological demands, role conflict, insecurity in university studies, and school–life conflict. WHO-5 = WHO Well-Being Index; Stress, Anxiety, and Depression = subscales of the DASS-21. ** *p* < 0.001.

**Table 4:** Invariance of the configural, metric, and scalar measurement of the 68-item CFPAU according to gender.

	χ^2^/gL	*p*-Value	CFI	TLI	RMSEA	Δχ^2^	pag	ΔCFI	ΔTLI	ΔRMSEA
***Dimension 1***										
Women	214.14/73	0.000	0.985	0.982	0.052					
Men	121.61/73	0.000	0.993	0.991	0.037					
Configural	335.76/146	0.000	0.988	0.985	0.046					
Metric	417.25/157	0.000	0.984	0.981	0.052	81.48/11	<0.0010	0.0043	0.004	0.006
Scalar	447.16/168	0.000	0.983	0.981	0.052	29.91/11	0.0016	0.001	0.000	0.0001
***Dimension 2***										
Women	581.55/184	0.000	0.940	0.931	0.055					
Men	471.4/184	0.000	0.940	0.931	0.056					
Configural	1053/368	0.000	0.939	0.931	0.055					
Metric	1100/386	0.000	0.937	0.931	0.055	47.12/18	0.0002	0.0026	−0.0004	−0.0002
Scalar	1202.5/404	0.000	0.929	0.926	0.057	102.39/18	<0.001	0.0074	0.0047	0.0019
***Dimension 3***										
Women	466.58/182	0.000	0.976	0.973	0.047					
Men	353.92/182	0.000	0.976	0.972	0.044					
Configural	820.55/364	0.000	0.976	0.972	0.045					
Metric	870.52/381	0.000	0.974	0.971	0.046	49.96/17	<0.001	0.0017	0.0007	0.0006
Scalar	916.58/398	0.000	0.972	0.971	0.046	46.06/17	<0.001	0.0015	0.0004	0.0003
***Dimension 4***										
Women	40.50/19	0.003	0.985	0.979	0.040					
Men	26/19	0.13	0.994	0.991	0.027					
Configural	66.5/38	0.000	0.989	0.984	0.035					
Metric	69.63/44	0.000	0.990	0.987	0.0311	3.13/6	0.7923	−0.0011	0.0035	−0.0042
Scalar	81.42/50	0.000	0.988	0.986	0.0323	11.78/6	0.066	0.0022	0.0010	0.0012
***Dimension 5***										
Women	0.649/2	0.723	1.00	1.00	00.00					
Men	1.12/2	0.57	1.00	1.00	00.00					
Configural	1.77/4		1.00	1.003	0.000					
Metric	2.12/7	0.95	1.00	1.003	0.000	0.34/3	0.95	0.0000	−0.0007	0.0000
Scalar	12.01/10	0.010	0.999	0.999	0.018	9.9/3	0.0008	0.0044	0.0183	0.0183

## Data Availability

The data presented in this study are available on request from the corresponding author due to ethical restrictions and institutional privacy policies concerning human participant data.

## Supplementary Materials

The following supporting information can be downloaded at: https://www.mdpi.com/article/10.3390/bs16060891/s1.
