# Supplementary material for: Psychometric Properties of the Questionnaire of Psychosocial Factors in University Environments
Source: Behav Sci (Basel). 2026 Jun 1;16(6):891. doi: 10.3390/bs16060891 (PMC13295542; doi:10.3390/bs16060891)
Supplement: Supplementary file 1 [file behavsci-16-00891-s001.zip › behavsci-4228409-supplementary.docx]

Annexes of the Questionnaire on Psychosocial Factors in University Environments (CFPAU)

**Annex A. Questionnaire on Psychosocial Factors in University Environments (74 items)**

**Overall Dimension 1**: Psychological Demands

Psychological demands have three components: quantitative; cognitive and sensory; and emotional labor, which are linked to the type of task performed. From a quantitative perspective, they refer to the volume of work in relation to the time available to complete it. The cognitive and sensory components focus on the level of mental demand required to carry out tasks or activities. Finally, psychological demands vary depending on whether or not one works with and for people; in such cases, emotional-type psychological demands are defined, which usually arise when working with people.

| **1.- Quantitative Psychological Demands** | | | |
| --- | --- | --- | --- |
| **Definition** | **Items** | **CFA factor loading** | **Source of risk** |
| Demands linked to academic activities that require a given work pace, as well as a high demand for time devoted to them.  They may become conflictive when there is more work than can be completed in the time devoted to university. | 1. Does studying at university require you to maintain a high work pace?  2. Do you think that at university assignments are given to you irregularly, causing you to feel overloaded with work?  3. Excluding class attendance, do you think the time you must devote to your academic activities is excessive? | 0.73  0.63  0.70 | Disorganization on the part of the professor or the student.  Poor time management.  Excessive assignment of activities to the student, with little or no planning of activities.  Excess of responsibilities (for example, working and studying).  Demands exceed the student’s capacity for action. |

| **2.- Cognitive Psychological Demands** | | | |
| --- | --- | --- | --- |
| **Definition** | **Items** | **CFA factor loading** | **Source of risk** |
| Demands related to mental processes (attention, creativity, decision-making) inherent to the university program being studied, as well as responsibility for one’s own actions. | 4. Does your academic program require you to handle many things at the same time?  5. Does your academic program require you to be able to propose new ideas?  6. Does your academic program require you to make decisions quickly?  7. Does your academic program require you to make difficult decisions?  8. Can the academic program you are studying have significant repercussions for your classmates, instructors, users, facilities, or society in general?  9. Does your academic program require you to manage a large amount of knowledge? | 0.75  Eliminated  0.59  0.68  Eliminated  Eliminated | Constantly requiring a high level of cognitive effort can wear students down.  Rather than depending on excessive demands, these refer to those that, for various reasons, exceed the student’s capacity.  Pursuing certain university programs may require greater cognitive effort or demand than others, due to the very nature of the field of study. |

| **3.- Emocional labor** | | | |
| --- | --- | --- | --- |
| **Definition** | **Items and polarity** | **CFA factor loading** | **Source of risk** |
| The self-regulation of emotions—that is, the inhibition of negative emotions and the forced expression of positive ones—implies a type of emotional labor necessary to maintain a neutral attitude toward the situations that arise.  Emotional demands require our ability to understand others’ circumstances, especially when those individuals express intense emotions, which may also constitute a type of psychosocial risk.  In the university environment, this risk may emerge particularly in programs that involve assisting individuals for whom certain changes are intended (for example: following a treatment, acquiring a skill, etc.). | 10. Are there moments or situations at the university in which it is difficult for you to manage your emotions?  11. Do you feel that studying and attending university is emotionally draining for you?  12. Do you find it hard to forget the university’s problems and/or demands?  13. Does your academic program require you to hide your emotions?  14. Do you feel that the university expects you to be someone you are not?  15. Do you feel that being a university student requires you to exert a great amount of emotional effort? | 0.80  0.74  0.78  0.71  0.80  Eliminated | The university imposes a high level of emotional demand on students, which may occasionally lead to confusion between their personal emotions and the emotions of those with whom they interact in the academic setting.  In the university and/or professional context, the profile of certain programs requires individuals to display or conceal particular emotions or attitudes, leading them to engage in emotionally exhausting efforts to do so. |

| **4.- Sensory Psychological Demands** | | | |
| --- | --- | --- | --- |
| **Definition** | **Items** | **CFA factor loading** | **Source of risk** |
| Demands related to the use of the senses (especially vision) and constant attentiveness to environmental stimuli, which require paying attention and remaining alert to details. These demands are directly linked to cognitive demands. | 16. Does your academic program require a high level of concentration?  17. Does your academic program require close or detailed observation?  18. Does your academic program require sustained attention?  19. Does your academic program require a high level of precision? | 0.75  0.74  0.89  Eliminated | High sensory demands associated with academic activities, which may worsen if environmental conditions or the student’s own characteristics are not adequate. |

In Table 1, the three retained factors of Dimension 1 are visually distinguished by color: quantitative and cognitive demands in green (WLS4), emotional labor in blue (WLS1), and sensory demands in purple (WLS2), corresponding to the final structure derived from the exploratory factor analysis.

**Table 1. Exploratory Factor Analysis Results for Dimension 1: Psychological Demands**

|  | **Emotional labor WLS1** | **Quantitative and cognitive demands WLS4** | **Sensory demands WLS2** | WLS3 | h2 | u2 | Com |
| --- | --- | --- | --- | --- | --- | --- | --- |
| CF1 | 0.14 | **0.65** | 0.03 | -0.05 | 0.56 | 0.44 | 1.1 |
| CF2 | 0.21 | **0.56** | -0.05 | -0.05 | 0.44 | 0.56 | 1.3 |
| CF3 | 0.19 | **0.61** | -0.04 | -0.07 | 0.5 | 0.5 | 1.2 |
| CF4 | 0.05 | **0.68** | 0.03 | 0.08 | 0.58 | 0.42 | 1 |
| **CF5** | 0.04 | 0 | -0.02 | 0.82 | 0.68 | 0.32 | 1 |
| CF6 | -0.04 | **0.5** | 0.08 | 0.35 | 0.53 | 0.47 | 1.9 |
| CF7 | 0.01 | **0.55** | 0.13 | 0.18 | 0.51 | 0.49 | 1.3 |
| **CF8** | -0.12 | 0.4 | 0.46 | 0.03 | 0.51 | 0.49 | 2.1 |
| **CF9** | 0.68 | 0.07 | 0.07 | 0.08 | 0.59 | 0.41 | 1.1 |
| CF10 | **0.84** | -0.03 | 0.04 | 0.01 | 0.69 | 0.31 | 1 |
| CF11 | **0.71** | 0.11 | -0.02 | -0.05 | 0.59 | 0.41 | 1.1 |
| CF12 | **0.59** | 0.12 | 0.05 | 0 | 0.48 | 0.52 | 1.1 |
| CF13 | **0.74** | 0.01 | -0.15 | 0.03 | 0.53 | 0.47 | 1.1 |
| CF14 | **0.8** | -0.02 | 0.11 | 0 | 0.67 | 0.33 | 1 |
| **CF15** | 0.07 | 0.1 | 0.73 | -0.07 | 0.64 | 0.36 | 1.1 |
| CF16 | 0.02 | 0 | **0.82** | 0.03 | 0.7 | 0.3 | 1 |
| CF17 | 0.05 | -0.1 | **0.9** | 0.03 | 0.76 | 0.24 | 1 |
| CF18 | -0.06 | 0.3 | **0.55** | -0.01 | 0.52 | 0.48 | 1.6 |
| **CF19** | 0.02 | 0.55 | 0.09 | 0.03 | 0.39 | 0.61 | 1.1 |

Note. Bolded items indicate those eliminated from the final model.

**Global Dimension 2:** Active Study and Opportunities

for Professional Development

Active study refers to a type of academic work that enables personal development through the degree of autonomy students possess to carry out the activities involved, as well as through the learning opportunities available to them—in other words, the concept of “control” within the previously mentioned Demand–Control–Social Support model. Additionally, the processes of identification, appropriation, and sense of belonging toward the university and the academic program constitute a key factor for individual development. These processes can also enhance students’ perception of support and control, thereby functioning as a protective factor.

| **1.- Control Over University Study Time** | | | |
| --- | --- | --- | --- |
| **Definition** | **Items** | **CFA factor loading** | **Source of risk** |
| The degree of autonomy and influence that individuals have regarding decision-making, schedules, courses, academic activities, and environmental conditions.  It includes control and flexibility over the time dedicated or assigned within the university context, as well as the possibility of taking breaks, and the pressure associated with not having enough time to complete personal and academic tasks. | 20. Do you control all the decisions that affect your studies at the university?  21. Do you have influence over the pace at which you complete your tasks and other academic activities?  22. Can you choose the people or classmates you work with during your classes?  23. Do you have influence over the schedule in which you attend the university (shift, days of the week, time you arrive and leave, etc.)?  24. Do you have influence over the professors who are assigned to you?  25. Do your professors allow you to use different methods to complete your tasks and other academic activities?  26. Is your opinion taken into account regarding the tasks or other academic activities assigned to you?  27. Is your opinion considered when changes are made to the places where you regularly study? (classrooms, laboratories, practice settings, or other off-campus locations)  28. Do you have influence over the order in which you complete your tasks and other academic activities?  29. While you are at the university, can you decide when to take a break? | 0.36  0.38  0.24  0.94  0.90  0.69  0.56  0.47  0.67  0.48 | Academic programs with highly structured curricula and complex content that require a demanding study pace.  Rigid or unclear academic planning by professors, with limited willingness to accept modifications.  Little or no flexibility in the assignment of shifts, courses, schedules, instructors, workspaces, rest periods, etc.  Low levels of student participation in decisions regarding their academic progression (order and pace of activities, methods used, peers they work with, and so forth). |

| **2.- Sense of Purpose and Professional Commitment** | | | |
| --- | --- | --- | --- |
| **Definition** | **Items** | **CFA factor loading** | **Source of risk** |
| Opportunities provided by the academic program and by the university as a whole to apply and develop the knowledge, skills, and attitudes necessary for competent professional practice.  This also includes the relationship established with the values or aims of the academic program, which may be associated with the social value and personal meaning of the profession.  A strong sense of purpose and professional commitment facilitates coping with the demands of the academic program. | 30. Are the activities you carry out as part of your professional training diverse?  31. Does the academic program you are studying require you to take initiative?  32. Does your academic program allow you to learn new things every day?  33. Does completing your tasks and other academic activities allow you to apply the skills and knowledge you have acquired?  34. Do the various activities you carry out at the university contribute significantly to your professional development?  35. Do the academic tasks and activities you perform seem important to you?  36. Do you feel committed to your profession or the academic program you are studying? | 0.80  0.79  0.79  0.41  0.99  0.98  0.47 | Higher risk is associated with academic programs containing monotonous or repetitive content and activities, or those that students perceive as irrelevant.  Programs involving complex and diverse activities may foster greater professional development, provided that students respond adequately to such demands.  Conversely, academic programs with which students feel little commitment may inhibit their sense of professional purpose and development.  Students may struggle to clearly understand the objectives of the tasks and activities they are required to complete, or may fail to perceive a social value or personal meaning in them.  This is related to the subdimension of control over university study time—lower control is associated with lower professional commitment. |

| **3.- Integration into the University** | | | |
| --- | --- | --- | --- |
| **Definition** | **Items** | **CFA factor loading** | **Source of risk** |
| The degree of identification, sense of belonging, and pride that students feel toward the institution in which they study.  This is a complementary dimension to sense of purpose and professional commitment. | 37. Would you like to work at the university where you study in the future?  38. Do you feel proud to belong to your university?  39. Do you feel that the problems your university faces are also your own?  40. Do you feel that your university is of great importance to you? | 0.77  0.44  0.84  0.87 | Perceiving the university as unrelated to students’ interests.  Low levels of identification, sense of belonging, or pride regarding the university as a whole (its mission, vision, values, and institutional identity).  Limited student involvement in university life and in the challenges faced by their institution. |

In table 2, the three retained factors of Dimension 2 are visually distinguished by color: control over work time at the university in blue (WLS2), professional sense and commitment in orange (WLS1), and integration into the university in green (WLS3), corresponding to the theoretically coherent three-factor solution retained in the EFA.

**Table 2. Exploratory Factor Analysis Results for Dimension 2:Active Study and Opportunities for Professional Development**

|  | **Professional sense and commitment**  **WLS1** | **Control over work time**  **WLS2** | **Integration into the university WLS3** | h2 | u2 | Com |
| --- | --- | --- | --- | --- | --- | --- |
| CF20 | 0.23 | **0.29** | -0.01 | 0.17 | 0.83 | 1.9 |
| CF21 | 0.22 | **0.25** | -0.04 | 0.13 | 0.87 | 2 |
| CF22 | 0.22 | **0.23** | 0.07 | 0.15 | 0.85 | 2.2 |
| CF23 | -0.18 | **0.57** | 0.03 | 0.32 | 0.68 | 1.2 |
| CF24 | -0.16 | **0.54** | 0.15 | 0.31 | 0.69 | 1.3 |
| CF25 | 0.31 | **0.37** | 0.03 | 0.3 | 0.7 | 2 |
| CF26 | 0.25 | **0.58** | 0.03 | 0.48 | 0.52 | 1.4 |
| CF27 | 0.09 | **0.53** | 0.03 | 0.33 | 0.67 | 1.1 |
| CF28 | 0.33 | **0.35** | -0.1 | 0.25 | 0.75 | 2.1 |
| CF29 | 0.03 | **0.54** | -0.04 | 0.3 | 0.7 | 1 |
| CF30 | **0.45** | 0.26 | 0.09 | 0.39 | 0.61 | 1.7 |
| CF31 | **0.31** | -0.09 | 0.07 | 0.11 | 0.89 | 1.3 |
| CF32 | **0.53** | -0.1 | 0.05 | 0.29 | 0.71 | 1.1 |
| CF33 | **0.72** | 0.09 | -0.04 | 0.54 | 0.46 | 1 |
| CF34 | **0.82** | 0.03 | 0.01 | 0.69 | 0.31 | 1 |
| CF35 | **0.72** | -0.04 | 0.12 | 0.58 | 0.42 | 1.1 |
| CF36 | **0.44** | -0.14 | 0.32 | 0.4 | 0.6 | 2.1 |
| CF37 | -0.11 | 0.22 | **0.49** | 0.28 | 0.72 | 1.5 |
| CF38 | 0.17 | -0.07 | **0.67** | 0.56 | 0.44 | 1.2 |
| CF39 | -0.16 | 0.13 | **0.52** | 0.26 | 0.74 | 1.3 |
| CF40 | 0.03 | 0.01 | **0.82** | 0.7 | 0.3 | 1 |

**Global Dimension 3:** Institutional Quality and Social Relationships

The university environment fosters interpersonal interaction, and these relationships—acting as protective factors and health-promoting conditions—support well-being: **social support** reflects its functional dimension, whereas a **sense of belonging** represents its emotional component. Being a student involves taking on roles that, depending on their clarity and coherence, may generate certainty or tension. In a changing environment, it is essential to anticipate and manage such changes in order to create a network of instrumental, emotional, and relational exchanges that facilitate adaptation. All of this occurs within a continuum of **demands, control, and coping resources** (such as social support), consistent with the theoretical model proposed **by Karasek (1979) and by Johnson and Hall (1988).**

| **1.- Predictability and Role Clarity** | | | |
| --- | --- | --- | --- |
| **Definition** | **Items** | **CFA factor loading** | **Source of risk** |
| Students are adequately, sufficiently, and timely informed about the activities or possible changes that may affect their studies and their progression through the university, allowing them to adapt appropriately.  This refers to concrete knowledge about the tasks to be carried out, objectives, responsibilities, expectations, rights, and freedoms associated with being a university student in a given institution. | 41. When you entered the university, were you clearly informed about how it operates (regulations, services, rights and responsibilities, etc.)?  42. Do you know what your rights and responsibilities are as a student?  43. Do you consider that your tasks and activities as a student have clear objectives?  44. Do you know exactly which tasks and activities are your responsibility as a student?  45. Do you know exactly what is expected of you as a student within the university? | 0.66  0.65  0.83  0.60  0.63 | Lack of predictability and clarity in the student role is associated with the absence of information, or with imprecise or insufficient information.  Limited or no knowledge among students regarding what is expected of them in their academic institution, including aspects such as regulations, university services, and student rights and responsibilities.  Lack of definition of student tasks and responsibilities.  This also relates to insufficient guidance and support for students during their progression through the university. |

| **2.- Role Conflict** | | | |
| --- | --- | --- | --- |
| **Definition** | **Items** | **CFA factor loading** | **Source of risk** |
| These are contradictory demands that arise within the university and that may generate professional, personal, or ethical conflicts when such demands differ from the students’ personal norms and values. | 46. Do you do things at school that are accepted by some people but not by others?  47. Are you required to carry out contradictory tasks or activities at the university?  48. Are you required to complete your academic tasks or activities in a specific way, even when you believe they could be done differently?  49. Do you have to perform academic tasks or activities that seem unnecessary to you? | 0.58  0.66  0.61  0.52 | Role conflict may occur when students are required to:  – Carry out tasks with which they disagree.  – Perform activities that are opposed, contradictory, or unnecessary.  – Complete tasks that are perceived as conflicting with their personal ethics or morals. |

| **3.- Social Support and Reinforcement at the University** | | | |
| --- | --- | --- | --- |
| **Definition** | **Items** | **CFA factor loading** | **Source of risk** |
| Receiving the necessary help—when required—from classmates and professors, as well as from institutional programs and support services, in order to adequately perform the academic tasks and activities demanded by the university.  It also involves promoting tasks and activities that increase opportunities for social interaction and facilitate support and reinforcement in collaborative work, as well as the establishment of meaningful social relationships. | 50. Do you receive feedback from your professors regarding your performance as a student?  51. Do you consider your classmates to be willing to listen to your problems and offer support if needed?  52. Do you consider your professors to be willing to listen to your problems and offer support if needed?  53. Are there institutional programs at your university that provide personal and/or academic support (psychopedagogical services, tutoring, psychological assistance, scholarships, etc.) that are applied effectively? | 0.48  0.65  0.64  Eliminated | Lack of support among classmates and from professors may stem from the absence of practices and/or activities that promote cooperation, interaction, and collaborative work, thereby fostering greater individual competitiveness and even isolation.  Deficient socialization processes among classmates and with other members of the university community.  Lack or insufficiency of institutional programs offering personal and/or academic support. |

| **4.- Sense of Belonging** | | | |
| --- | --- | --- | --- |
| **Definition** | **Items and polarity** | **CFA factor loading** | **Source of risk** |
| The possibility of forming relationships with classmates at the university, expressed through communication and mutual support in carrying out academic activities, as well as the sense of belonging to a group. | 54. Do you consider the university to be a space with potential for establishing lasting social relationships?  55. Is there a good atmosphere between you and your classmates at the university?  56. Do classmates support one another in carrying out their activities?  57. At the university, do you feel that you are part of a community—whether at the university level, within your academic program, or within your class group? | 0.75  0.73  0.82  0.72 | A sense of belonging deteriorates when the institution or the activities required of students promote isolation and interpersonal competitiveness, which negatively affects the quality of relationships and the overall university environment.  Students may fail—or may have no interest—in developing social relationships or in feeling part of the community to which they belong. |

| **5.- Institutional Quality** | | | |
| --- | --- | --- | --- |
| **Definition** | **Items** | **CFA factor loading** | **Source of risk** |
| This refers to the quality of attention, accessibility, and institutional management, as well as the importance that professors and administrative staff place on students’ needs. | **Your professors and/or administrative staff:**  58. Do they ensure that you have good opportunities for academic and professional development?  59. Do they care about ensuring that you feel comfortable and satisfied with the university?  60. Do they provide appropriate solutions to the problems you encounter?  61. Do they maintain good communication with you and your classmates?  62. Are they approachable and accessible to students? | **0.80**  **0.81**  **0.82**  **0.75**  **0.75** | Deficiencies in the attention, accessibility, and/or management of situations reported by students.  Students perceive that they are not listened to when they ask for help or express a concern.  Poor communication from professors or administrative personnel toward students. |

In table 3, the four retained factors of Dimension 3 are visually distinguished by color: predictability and role clarity in blue (WLS4), role conflict in orange (WLS2), sense of belonging in purple (WLS3), and institutional quality in green (WLS1), corresponding to the theoretically coherent structure retained after eliminating the single-item fifth factor.

**Table 3. Exploratory Factor Analysis Results for Dimension 3:Institutional Quality and Social Relationships**

|  | **Institutional quality WLS1** | **Sense of belonging**  **WLS3** | **Predictability and role clarity WLS4** | **Role conflict WLS2** | WLS5 | h2 | u2 | Com |
| --- | --- | --- | --- | --- | --- | --- | --- | --- |
| CF41 | 0.11 | 0.05 | 0.55 | -0.06 | -0.15 | 0.39 | 0.61 | 1.3 |
| CF42 | 0.04 | 0.06 | 0.73 | -0.03 | -0.21 | 0.57 | 0.43 | 1.2 |
| CF43 | 0.15 | -0.01 | 0.66 | 0.01 | 0.09 | 0.57 | 0.43 | 1.1 |
| CF44 | -0.09 | 0.02 | 0.77 | 0.06 | 0.12 | 0.58 | 0.42 | 1.1 |
| CF45 | -0.01 | 0.02 | 0.64 | -0.05 | 0.14 | 0.48 | 0.52 | 1.1 |
| CF46R | 0.02 | 0.07 | -0.18 | 0.59 | -0.02 | 0.4 | 0.6 | 1.2 |
| CF47R | -0.09 | 0.09 | 0.03 | 0.66 | 0.08 | 0.43 | 0.57 | 1.1 |
| CF48R | 0.03 | -0.01 | -0.06 | 0.68 | -0.07 | 0.49 | 0.51 | 1 |
| CF49R | 0.11 | -0.09 | 0.13 | 0.71 | 0.02 | 0.52 | 0.48 | 1.2 |
| CF50 | **0.36** | -0.05 | 0.21 | -0.2 | 0.11 | 0.31 | 0.69 | 2.5 |
| CF51 | 0.19 | **0.47** | 0.04 | -0.13 | 0.06 | 0.4 | 0.6 | 1.6 |
| CF52 | **0.5** | 0.14 | 0.01 | -0.08 | 0.18 | 0.44 | 0.56 | 1.5 |
| **CF53** | **0.28** | **0.07** | **0.13** | **0** | **0.42** | **0.42** | **0.58** | **2** |
| CF54 | 0.02 | **0.47** | 0.09 | -0.02 | 0.28 | 0.42 | 0.58 | 1.7 |
| CF55 | -0.08 | **0.91** | 0.01 | 0.05 | -0.04 | 0.77 | 0.23 | 1 |
| CF56 | 0.09 | **0.82** | -0.03 | -0.03 | 0 | 0.74 | 0.26 | 1 |
| CF57 | 0.09 | **0.65** | 0.08 | 0.01 | 0.02 | 0.53 | 0.47 | 1.1 |
| CF58 | **0.75** | -0.01 | 0.14 | 0.02 | -0.05 | 0.65 | 0.35 | 1.1 |
| CF59 | **0.85** | -0.04 | 0.02 | 0.02 | -0.02 | 0.71 | 0.29 | 1 |
| CF60 | **0.86** | -0.01 | -0.01 | 0.04 | -0.03 | 0.72 | 0.28 | 1 |
| CF61 | **0.78** | 0.07 | -0.03 | 0 | 0.03 | 0.64 | 0.36 | 1 |
| CF62 | **0.78** | 0.08 | -0.06 | 0 | 0.05 | 0.65 | 0.35 | 1 |

Note. Bolded items indicate those eliminated from the final model.

**Global Dimension 4:** Recognition and Certainty in the Career

This dimension refers primarily to the recognition students receive from their school for the effort they invest in their academic, athletic, and/or cultural activities, as well as the degree of security they feel regarding the possibility of continuing their studies—without temporary or permanent interruption—and their ability to keep participating in all university-related activities.

| **1.- Recognition** | | | |
| --- | --- | --- | --- |
| **Definition** | **Items** | **CFA factor loading** | **Source of risk** |
| Recognition from classmates, professors, or administrative staff for the effort invested in academic, athletic, and/or cultural activities. | 63. My professors give me the recognition I deserve.  64. My classmates at the university give me the recognition I deserve.  65. When I think about all the tasks and activities (academic, athletic, and/or cultural) I have completed at the university, I feel that I DO receive appropriate recognition. | 0.81  0.79  0.79 | Recognition from classmates, professors, and/or the university as a whole is perceived as low or nonexistent regarding the student’s achievements.  Perception of being treated unfairly or improperly. |

| **2.- Insecurity Regarding University Studies** | | | |
| --- | --- | --- | --- |
| **Definition** | **Items** | **CFA factor loading** | **Source of risk** |
| Concerns related to the permanent interruption of studies, the mandatory assignment of new activities or courses, or modifications to the academic workload.  It also includes worries about the financial resources needed to continue studying, as well as the possibility of not completing the degree and obtaining the professional title. | **How worried are you about:**  66. …how difficult it would be to resume your studies if you had to interrupt them?  67. …being assigned courses or other academic activities against your will?  68. …having your schedule changed (shift, days of the week, start and end times) against your will?  69. …losing the financial benefits you currently rely on to pursue your studies (family support, scholarships, in-kind assistance, etc.)?  70. …not finishing your academic career and obtaining your professional degree? | **0.65**  **0.72**  **0.67**  **0.72**  **0.66** | Inability to continue studies due to economic, personal, family, or other factors, as well as the social and academic consequences associated with this.  Difficulty adapting to imposed changes in schedules, courses, and/or academic activities.  Economic instability or financial shortages that threaten academic continuity.  Insecurity or uncertainty about completing the academic career and obtaining the university degree. |

In table 4, the two retained factors of Dimension 4 are visually distinguished by color: recognition in blue (WLS2) and insecurity in university studies in orange (WLS1), corresponding to the theoretically coherent two-factor solution retained in the EFA.

**Table 4. Exploratory Factor Analysis Results for Dimension 4:Recognition and Certainty in the Career**

|  | Insecurity in university studies **WLS1** | Recognition **WLS2** | h2 | u2 | com |
| --- | --- | --- | --- | --- | --- |
| CF63 | -0.01 | 0.77 | 0.59 | 0.41 | 1 |
| CF64 | 0.02 | 0.71 | 0.50 | 0.50 | 1 |
| CF65 | 0.00 | 0.79 | 0.63 | 0.37 | 1 |
| CF66R | 0.65 | -0.09 | 0.43 | 0.57 | 1 |
| CF67R | 0.71 | 0.02 | 0.51 | 0.49 | 1 |
| CF68R | 0.68 | 0.00 | 0.47 | 0.53 | 1 |
| CF69R | 0.66 | 0.03 | 0.44 | 0.56 | 1 |
| CF70R | 0.70 | 0.02 | 0.49 | 0.51 | 1 |

**Global dimension 5:** School-Life Conflict

This dimension refers to the need to respond to simultaneous or overlapping demands from the university environment and from personal, family, and/or work domains, which may generate negative effects on students’ health and well-being.

| **1. School-Life Conflict** | | | |
| --- | --- | --- | --- |
| **Definition** | **Items** | **CFA factor loading** | **Source of risk** |
| The presence of simultaneous demands from the university environment and from personal, family, and/or work domains, which may generate significant conflicts for students. | 71. There are moments when I need to be at the university and at home or at work at the same time.  72. I feel that my academic activities consume so much time or energy that they affect my personal, family, and/or work life.  73. The demands of the university interfere with my personal, family, and/or work life.  74. At times, I must change my personal, family, and/or work plans because of the demands of the university. | 0.52  0.83  0.90  0.80 | Occasional or constant incompatibility between university demands and personal, family, and/or work responsibilities.  High time or energy demands from the university that interfere with other areas of life.  Simultaneous demands emerging from the university and one or more other domains (personal, family, and/or work). |

In table 5, Dimension 5 is represented as a single-factor structure corresponding to school–life conflict (WLS1), comprising items 71–74, which was retained due to its theoretical relevance and adequate internal consistency.

**Table 5. Exploratory Factor Analysis Results for Dimension 5: School-Life Conflict**

| **Ítem** | **School- life conflict. WLS1** | h2 | u2 |
| --- | --- | --- | --- |
| CF71 | 0.62 | 0.39 | 0.61 |
| CF72 | 0.88 | 0.77 | 0.23 |
| CF73 | 0.91 | 0.84 | 0.16 |
| CF74 | 0.79 | 0.63 | 0.37 |

**These items were eliminated in the final version of the CFPAU.**

| **Dimension** | **Eliminated Items** | **Observation** |
| --- | --- | --- |
| 1. Psychological Demands | 5, 8, 9, 15, 19 | Eliminated ítems |
| 2. Active Study and Opportunities for Professional Development | — | No eliminations reported |
| 3. Institutional Quality and Social Relationships | 53 | Eliminated item |
| 4. Recognition and Certainty in the Career | — | No eliminations reported |
| 5. School-Life Conflict | — | No eliminations reported |

**Supplementary Table S6**

CFPAU items are scored using a five-point response format coded as 0, 25, 50, 75, and 100, where higher scores indicate a greater presence of the evaluated psychosocial factor.

*Item-Level Descriptive Statistics and Distributional Properties of the CFPAU (N = 1,221)*

| **Item** | **M** | **SD** | **Skewness** | **Kurtosis** | **Missing (%)** |
| --- | --- | --- | --- | --- | --- |
| **CF1** | 62.30 | 22.69 | -0.127 | -0.163 | 0.00 |
| **CF2** | 55.73 | 23.43 | 0.097 | -0.151 | 0.00 |
| **CF3** | 59.59 | 26.42 | -0.158 | -0.571 | 0.00 |
| **CF4** | 64.64 | 26.42 | -0.281 | -0.675 | 0.00 |
| **CF5** | 71.74 | 23.37 | -0.491 | -0.359 | 0.00 |
| **CF6** | 69.57 | 23.73 | -0.378 | -0.426 | 0.00 |
| **CF7** | 64.07 | 27.28 | -0.229 | -0.844 | 0.00 |
| **CF8** | 82.84 | 20.05 | -0.967 | 0.361 | 0.00 |
| **CF9** | 52.62 | 29.04 | 0.012 | -0.823 | 0.00 |
| **CF10** | 50.96 | 29.63 | 0.020 | -0.841 | 0.00 |
| **CF11** | 51.11 | 29.68 | 0.076 | -0.900 | 0.00 |
| **CF12** | 35.52 | 32.55 | 0.508 | -0.842 | 0.00 |
| **CF13** | 30.02 | 30.69 | 0.730 | -0.491 | 0.00 |
| **CF14** | 50.18 | 30.63 | 0.039 | -0.939 | 0.00 |
| **CF15** | 79.59 | 22.73 | -0.892 | 0.082 | 0.00 |
| **CF16** | 83.68 | 20.93 | -1.175 | 0.842 | 0.00 |
| **CF17** | 84.58 | 19.82 | -1.168 | 0.860 | 0.00 |
| **CF18** | 74.94 | 24.66 | -0.620 | -0.526 | 0.00 |
| **CF19** | 60.59 | 30.02 | -0.284 | -0.862 | 0.00 |
| **CF20** | 71.66 | 23.47 | -0.522 | -0.351 | 0.00 |
| **CF21** | 69.27 | 24.50 | -0.516 | -0.192 | 0.00 |
| **CF22** | 72.11 | 21.19 | -0.447 | 0.060 | 0.00 |
| **CF23** | 34.28 | 36.21 | 0.615 | -1.043 | 0.00 |
| **CF24** | 28.25 | 34.78 | 0.863 | -0.645 | 0.00 |
| **CF25** | 68.22 | 24.39 | -0.454 | -0.325 | 0.00 |
| **CF26** | 56.77 | 27.04 | -0.187 | -0.545 | 0.00 |
| **CF27** | 47.63 | 31.06 | 0.005 | -0.963 | 0.00 |
| **CF28** | 70.50 | 26.22 | -0.637 | -0.172 | 0.00 |
| **CF29** | 43.90 | 30.60 | 0.253 | -0.829 | 0.00 |
| **CF30** | 64.44 | 22.89 | -0.127 | -0.408 | 0.00 |
| **CF31** | 80.67 | 21.43 | -0.858 | -0.005 | 0.00 |
| **CF32** | 87.78 | 19.14 | -1.543 | 1.887 | 0.00 |
| **CF33** | 73.57 | 21.99 | -0.524 | -0.257 | 0.00 |
| **CF34** | 76.25 | 22.15 | -0.677 | -0.060 | 0.00 |
| **CF35** | 74.80 | 22.59 | -0.530 | -0.380 | 0.00 |
| **CF36** | 82.58 | 21.75 | -1.171 | 0.859 | 0.00 |
| **CF37** | 49.49 | 33.25 | 0.017 | -1.104 | 0.00 |
| **CF38** | 75.82 | 25.57 | -0.910 | 0.256 | 0.00 |
| **CF39** | 40.95 | 31.23 | 0.243 | -0.952 | 0.00 |
| **CF40** | 70.07 | 26.95 | -0.560 | -0.466 | 0.00 |
| **CF41** | 68.73 | 31.08 | -0.723 | -0.501 | 0.00 |
| **CF42** | 66.99 | 28.29 | -0.578 | -0.420 | 0.00 |
| **CF43** | 70.41 | 22.53 | -0.424 | -0.139 | 0.00 |
| **CF44** | 77.86 | 23.14 | -0.928 | 0.411 | 0.00 |
| **CF45** | 71.83 | 24.64 | -0.714 | 0.108 | 0.00 |
| **CF46R** | 50.80 | 30.75 | 0.085 | -0.914 | 0.00 |
| **CF47R** | 71.92 | 30.57 | -0.801 | -0.468 | 0.00 |
| **CF48R** | 46.38 | 27.32 | 0.131 | -0.532 | 0.00 |
| **CF49R** | 44.86 | 26.93 | 0.096 | -0.509 | 0.00 |
| **CF50** | 56.39 | 27.01 | -0.126 | -0.606 | 0.00 |
| **CF51** | 62.69 | 29.33 | -0.473 | -0.608 | 0.00 |
| **CF52** | 62.28 | 26.63 | -0.347 | -0.515 | 0.00 |
| **CF53** | 73.03 | 25.59 | -0.690 | -0.185 | 0.00 |
| **CF54** | 73.79 | 23.72 | -0.744 | 0.178 | 0.00 |
| **CF55** | 73.26 | 24.95 | -0.820 | 0.267 | 0.00 |
| **CF56** | 70.62 | 24.78 | -0.608 | -0.095 | 0.00 |
| **CF57** | 72.63 | 26.39 | -0.864 | 0.238 | 0.00 |
| **CF58** | 69.41 | 22.15 | -0.418 | -0.047 | 0.00 |
| **CF59** | 63.72 | 25.67 | -0.376 | -0.331 | 0.00 |
| **CF60** | 63.31 | 25.35 | -0.392 | -0.256 | 0.00 |
| **CF61** | 69.55 | 23.76 | -0.448 | -0.227 | 0.00 |
| **CF62** | 67.22 | 24.31 | -0.447 | -0.197 | 0.00 |
| **CF63** | 62.30 | 23.09 | -0.263 | -0.125 | 0.00 |
| **CF64** | 61.68 | 25.28 | -0.429 | -0.121 | 0.00 |
| **CF65** | 61.93 | 25.19 | -0.363 | -0.146 | 0.00 |
| **CF66R** | 32.70 | 29.55 | 0.721 | -0.287 | 0.00 |
| **CF67R** | 38.88 | 30.45 | 0.443 | -0.732 | 0.00 |
| **CF68R** | 34.17 | 30.55 | 0.604 | -0.574 | 0.00 |
| **CF69R** | 32.41 | 32.88 | 0.754 | -0.559 | 0.00 |
| **CF70R** | 20.35 | 28.58 | 1.364 | 0.945 | 0.00 |
| **CF71** | 46.45 | 28.43 | 0.122 | -0.573 | 0.00 |
| **CF72** | 54.35 | 27.95 | -0.079 | -0.681 | 0.00 |
| **CF73** | 51.10 | 28.75 | -0.015 | -0.709 | 0.00 |
| **CF74** | 57.51 | 28.61 | -0.228 | -0.697 | 0.00 |

*Note.* N = 1,221. M = mean; SD = standard deviation. Items marked with 'R' are reverse-scored. Missing data percentage is 0.00 for all items, indicating complete data. Skewness and kurtosis values within the range of ±2 indicate acceptable univariate normality (Hair et al., 2010). No response categories were collapsed; all original 5-point response options were retained for all analyses.

**Supplementary Table S7**

*Spearman Correlations Between CFPAU Subscales and External Validity Measures (WHO-5 and DASS-21) by Dimension*

**Dimension 1: Psychological Demands**

| **Subscale / Measure** | **M (SD)** | **1** | **2** | **3** | **4** | **5** | **6** |
| --- | --- | --- | --- | --- | --- | --- | --- |
| **1. Quantitative-Cognitive Demands** | 62.65 ± 18.80 | -- |  |  |  |  |  |
| **2. Emotional Labor** | 43.56 ± 23.06 | .582** | -- |  |  |  |  |
| **3. Sensory Demands** | 81.06 ± 19.07 | .535** | .268** | -- |  |  |  |
| **4. WHO-5** | 58.53 ± 21.73 | -.119** | -.273** | -.097** | -- |  |  |
| **5. Stress** | 17.98 ± 11.10 | .222** | .401** | .142** | -.578** | -- |  |
| **6. Anxiety** | 13.35 ± 10.70 | .210** | .448** | .096** | -.414** | .800** | -- |
| **7. Depression** | 14.16 ± 10.90 | .184** | .460** | .060* | -.528** | .811** | .795** |

**Dimension 2: Active Work and Professional Development**

| **Subscale / Measure** | **M (SD)** | **1** | **2** | **3** | **4** | **5** | **6** |
| --- | --- | --- | --- | --- | --- | --- | --- |
| **1. Control over work time** | 56.25 ± 15.72 | -- |  |  |  |  |  |
| **2. Professional sense & commitment** | 77.15 ± 14.70 | .329** | -- |  |  |  |  |
| **3. Integration into university** | 59.08 ± 21.63 | .270** | .394** | -- |  |  |  |
| **4. WHO-5** | 58.53 ± 21.73 | .234** | .117** | .190** | -- |  |  |
| **5. Stress** | 17.98 ± 11.10 | -.162** | -.056 | -.041 | -.578** | -- |  |
| **6. Anxiety** | 13.35 ± 10.70 | -.086** | -.095** | .010 | -.414** | .800** | -- |
| **7. Depression** | 14.16 ± 10.90 | -.086** | -.129** | -.072* | -.528** | .811** | .795** |

**Dimension 3: Institutional Quality and Social Relations**

| **Subscale / Measure** | **M (SD)** | **1** | **2** | **3** | **4** | **5** | **6** |
| --- | --- | --- | --- | --- | --- | --- | --- |
| **1. Predictability & role clarity** | 71.16 ± 19.84 | -- |  |  |  |  |  |
| **2. Role conflict** | 53.47 ± 21.39 | -.026 | -- |  |  |  |  |
| **3. Sense of belonging** | 70.59 ± 20.39 | .376** | .028 | -- |  |  |  |
| **4. Institutional quality** | 64.55 ± 19.19 | .517** | .029 | .535** | -- |  |  |
| **5. WHO-5** | 58.53 ± 21.73 | .195** | -.058* | .238** | .293** | -- |  |
| **6. Stress** | 17.98 ± 11.10 | -.109** | -.131** | -.158** | -.185** | -.578** | -- |
| **7. Anxiety** | 13.35 ± 10.70 | -.090** | -.249** | -.152** | -.131** | -.414** | .800** |
| **8. Depression** | 14.16 ± 10.90 | -.094** | -.189** | -.184** | -.135** | -.521** | .810** |

**Dimension 4: Recognition and Certainty in the Career**

| **Subscale / Measure** | **M (SD)** | **1** | **2** | **3** | **4** | **5** |
| --- | --- | --- | --- | --- | --- | --- |
| **1. Recognition** | 61.96 ± 21.02 | -- |  |  |  |  |
| **2. Insecurity in university studies** | 31.70 ± 23.00 | -.063* | -- |  |  |  |
| **3. WHO-5** | 58.53 ± 21.73 | .335** | .100** | -- |  |  |
| **4. Stress** | 17.98 ± 11.10 | -.212** | -.199** | -.578** | -- |  |
| **5. Anxiety** | 13.35 ± 10.70 | -.171** | -.195** | -.414** | .800** | -- |
| **6. Depression** | 14.16 ± 10.90 | -.200** | -.152** | -.528** | .811** | .795** |

**Dimension 5: School-Life Conflict**

| **Subscale / Measure** | **M (SD)** | **1** | **2** | **3** | **4** | **5** |
| --- | --- | --- | --- | --- | --- | --- |
| **1. School-life conflict** | — | -- |  |  |  |  |
| **2. WHO-5** | 58.53 ± 21.73 | -.147** | -- |  |  |  |
| **3. Stress** | 17.98 ± 11.10 | .313** | -.578** | -- |  |  |
| **4. Anxiety** | 13.35 ± 10.70 | .331** | -.414** | .800** | -- |  |
| **5. Depression** | 14.16 ± 10.90 | .307** | -.528** | .811** | .795** | -- |

*Note.* Correlations are Spearman's rho. WHO-5 = WHO Well-Being Index; Stress, Anxiety, and Depression = subscales of the DASS-21. *p < .05. **p < .001.
